# Supplementary material for: Polyketide synthase gene clusters in the ericoid mycorrhizal fungus Oidiodendron maius: genome-wide prediction, expression profiling, and functional characterization of knockout mutants
Source: Front Plant Sci. 2026 Sep 16;17:1924430. doi: 10.3389/fpls.2026.1924430 (PMC13623903; doi:10.3389/fpls.2026.1924430)
Supplement: Supplementary file 1 [file SupplementaryFile1.zip › Supplementary Materials/Supplementary Folder 1.zip!/Oidma1_AssemblyScaffolds_Repeatmasked/knownclusterblast/cluster55/jgi.p_Oidma1_46487_mibig_hits.html]

| MIBiG Protein | Description | MIBiG Cluster | MiBiG Product | % ID | % Coverage | BLAST Score | E-value |
| --- | --- | --- | --- | --- | --- | --- | --- |
| AGO86666.1 | putative\_MFS\_transporter | BGC0001255 | Equisetin\_biosynthetic\_gene\_cluster | 44 | 95.4813359528 | 395 | 1e-109 |
| AGO65989.1 | putative\_MFS\_transporter | BGC0000992 | Fusaridione\_A\_biosynthetic\_gene\_cluster | 44 | 93.9096267191 | 383 | 5.3e-106 |
| ACD39772.1 | major\_facilitator\_superfamily\_transporter | BGC0000134 | Radicicol\_biosynthetic\_gene\_cluster | 39 | 95.0884086444 | 360 | 3.7e-99 |
| KFH48645.1 | putative\_transporter\_-like\_protein | BGC0000317 | Cephalosporin\_biosynthetic\_gene\_cluster | 41 | 92.5343811395 | 359 | 6.3e-99 |
| BAK26557.1 | putative\_MFS\_multidrug\_transporter | BGC0000977 | Cyclopiazonic\_acid\_biosynthetic\_gene\_cluster | 40 | 93.3202357564 | 352 | 7.7e-97 |
| ADI24938.1 | VrtL | BGC0000168 | Viridicatumtoxin\_biosynthetic\_gene\_cluster | 37 | 96.0707269155 | 302 | 9.2e-82 |
| CAP95407.1 |  | BGC0001404 | Sorbicillin\_biosynthetic\_gene\_cluster | 33 | 91.3555992141 | 243 | 3.9e-64 |
| XP\_011392678.1 | hypothetical\_protein | BGC0001281 | Ustilagic\_acid\_biosynthetic\_gene\_cluster | 26 | 74.2632612967 | 149 | 1.3e-35 |
| AMY15073.1 | MFS\_transporter | BGC0001339 | Squalestatin\_S1\_biosynthetic\_gene\_cluster | 25 | 88.2121807466 | 116 | 9.3e-26 |
| ANF07284.1 | MFS | BGC0001340 | Byssochlamic\_acid\_biosynthetic\_gene\_cluster | 24 | 77.7996070727 | 87 | 4.6e-17 |
| ABC87523.1 | putative\_drug\_efflux\_transporter | BGC0001011 | Meridamycin\_biosynthetic\_gene\_cluster | 25 | 37.1316306483 | 59 | 1.3e-08 |
| AAK06799.1 | putative\_export\_protein\_SimEx | BGC0001072 | Simocyclinone\_biosynthetic\_gene\_cluster | 24 | 34.5776031434 | 59 | 1.8e-08 |
| AAL15595.1 | Sim17 | BGC0000270 | Simocyclinone\_biosynthetic\_gene\_cluster | 24 | 34.5776031434 | 58 | 2.3e-08 |
| AHW57792.1 | PgaJ3 | BGC0000262 | Rabelomycin\_biosynthetic\_gene\_cluster | 29 | 22.2003929273 | 58 | 3e-08 |
| EHM27501.1 | putative\_export\_protein | BGC0000235 | Kiamycin\_biosynthetic\_gene\_cluster | 27 | 31.8271119843 | 56 | 1.5e-07 |
| WP\_016325506.1 | hypothetical\_protein | BGC0001283 | Arsenopolyketides\_biosynthetic\_gene\_cluster | 22 | 37.5245579568 | 52 | 2.2e-06 |
